# Fluoxetine-enhanced autophagy ameliorates early brain injury via inhibition of NLRP3 inflammasome activation following subrachnoid hemorrhage in rats

**DOI:** 10.1186/s12974-017-0959-6

**Published:** 2017-09-13

**Authors:** Jian-ru Li, Hang-zhe Xu, Sheng Nie, Yu-cong Peng, Lin-Feng Fan, Zhi-jiang Wang, Cheng Wu, Feng Yan, Jing-yin Chen, Chi Gu, Chun Wang, Jing-sen Chen, Lin Wang, Gao Chen

**Affiliations:** 1 Department of Neurosurgery, The Second Affiliated Hospital of Zhejiang University School of Medicine, Hangzhou, Zhejiang China; 2 Department of Neurosurgery, Sir Run Run Shaw Hospital, Zhejiang University School of Medicine, Hangzhou, Zhejiang China

**Keywords:** Subarachnoid hemorrhage, NLRP3 inflammasome, Fluoxetine, Inflammation, Early brain injury, Autophagy

## Abstract

**Background:**

The NLRP3 inflammasome is a multiprotein complex that regulates the innate immune inflammatory response by activating caspase-1 and subsequent IL-1β and IL-18. Fluoxetine has been shown to have the anti-inflammatory properties in many disease models. However, the effects and mechanisms of these effects of fluoxetine in early brain injury after subarachnoid hemorrhage (SAH) have not been defined.

**Methods:**

The SAH model was induced by an endovascular perforation in adult male Sprague-Dawley (SD) rats weighing 300–320 g. N-Ac-Tyr-Val-Ala-Asp-chloromethyl ketone (AC-YVAD-CMK) was injected intraperitoneally (5 mg/kg) 1 h after SAH. Fluoxetine was administered via intravenous route 6 h after SAH. 3-Methyladenine (3-MA) was intracerebroventricularly injected 20 min before SAH. SAH grade, neurological function, brain water content, propidium iodide (PI) staining, western blot, double immunostaining, and transmission electron microscopy were performed.

**Results:**

Expression of caspase-1 increased and peaked at 24 h after SAH. Caspase activation was along with the increased necrotic cells, which occurred mainly in neurons. Necrotic cell death of microglia and astrocyte were also found. Administration of AC-YVAD-CMK, a caspase-1 inhibitor, reduced the expression of IL-1β and IL-18 and the number of PI-positive cells, attenuated brain edema, and improved neurological function, which was also observed in fluoxetine-treated rats. Furthermore, fluoxetine treatment significantly decreased the expression of NLRP3 and cleaved caspase-1 and upregulated the expression of beclin-1, a marker for autophagy. Finally, the effects of fluoxetine in NLRP3 inflammasome activation were reversed by additional 3-MA administration.

**Conclusions:**

Together, our present study indicated that NLRP3 inflammasome and caspase-1 activation play a deleterious role in early brain injury and fluoxetine mitigates NLRP3 inflammasome and caspase-1 activation through autophagy activation after SAH, providing a potential therapeutic agent for SAH treatment.

**Electronic supplementary material:**

The online version of this article (10.1186/s12974-017-0959-6) contains supplementary material, which is available to authorized users.

## Background

Subarachnoid hemorrhage (SAH) refers to a consequence of bleeding within the subarachnoid space. Cerebral aneurysm rupture is the most frequent etiology of SAH. Although SAH accounts for only 5% of stroke cases, the mortality rate could be as high as 67% in the first few months [1, 2]. The significant mortality and morbidity may result from both the early brain injury and delayed cerebral ischemia [1]. Therapies targeted toward delayed cerebral ischemia have had limited success, which has led to a focus on mechanisms of early brain injury [3, 4]. In the past few decades, many pathological mechanisms of early brain injury have been proposed. A ruptured aneurysm leads to many physiological derangements such as elevated intracranial pressure (ICP), decreased cerebral blood flow (CBF), and decreased cerebral perfusion pressure (CCP). These events initiate various cascades of injuries such as inflammation, oxidative stress, blood brain barrier dysfunction, brain edema, and apoptosis [5, 6].

The inflammasome is a multiprotein complex that regulate the innate immune inflammatory response. Several inflammasomes, such as NLRP1, NLRP3, and AIM2, have been described in the central nervous system(CNS) and associated with brain injury [7, 8]. Among them, NLRP3 inflammasome is the most studied one, especially in SAH [9, 10]. Inflammasome activation triggers activation of caspase-1 and mature of inflammatory cytokines, such as IL-1β and IL-18, activate the immune response eventually [11]. In addition, caspase-1 activation also induces the regulated necrotic cell death, which is characterized by cell membrane rupture and subsequent inflammation [12]. Accumulating evidence have demonstrated that inhibiting NLRP3 inflammasome activation attenuates neuroinflammation and cell death in early brain injury and improves neurological function following SAH [9, 10, 13]. Moreover, recent studies have uncovered that autophagy activation inhibited inflammasomes activation [14–16]. Fluoxetine, a selective serotonin reuptake inhibitor (SSRI), is widely used to treat depression, obsessive-compulsive disorder, bulimia, and panic disorder [17]. Recent studies have indicated that fluoxetine exhibits beneficial effects in neurological disorders, including ischemic stroke [18], brain injury [19], and spinal cord injury [20]. In fact, it is reported that fluoxetine induces the autophagy pathway through FK506 binding protein 51(FKBP51) [21]. Furthermore, fluoxetine inhibits NLRP3 inflammasome activation in depression [22]. However, the effect of fluoxetine on NLRP3 inflammasome activation and potential mechanisms in early brain injury after SAH remains unclear.

In the present study, we investigated the role of NLRP3 inflammasome and caspase-1 activation in early brain injury after SAH and the effect of fluoxetine on NLRP3 inflammasome and caspase-1 activation and the underlying possible mechanisms in early brain injury after SAH. Our findings indicated that caspase-1 activation was induced at the early stages of SAH and promoted necrotic cell death and expression of inflammatory cytokines (IL-1β and IL-18) in early brain injury after SAH. In addition, our study showed that fluoxetine reduces NLRP3 inflammasome and caspase-1 activation in early brain injury after SAH, at least partly, by activating autophagy, providing potential therapeutic interventions for SAH.

## Methods

### Animals

Adult male Sprague-Dawley (SD) rats were purchased from Slac Laboratory Animal Company, Shanghai, China. The rats were housed in a controlled temperature and humidity conditions with a 12-h light/dark cycle.

### Study design

The experiment was designed as follows (Additional file 1: Figure S1).

#### Experiment 1

Forty-eight rats were randomly divided into two groups: sham and SAH group. Western blot, propidium iodide (PI) staining, and transmission electron microscopy (TEM) were performed.

#### Experiment 2

Forty-three rats were randomly divided into the following four groups: sham, sham + AC-YVAD-CMK, SAH + vehicle, and SAH + AC-YVAD-CMK group.Western blot and PI staining were performed at 24 h after SAH. SAH grade and neurological scores were blindly assessed at the same time.

#### Experiment 3

Eighty-six rats were randomly assigned into the following four groups: sham, sham + Fluoxetine (Fluo), SAH + vehicle, and SAH + Fluo group. PI staining, double immunostaining, western blot, and brain water content were measured at 24 h after SAH. Neurological scores were also blindly assessed at the same time.

#### Experiment 4

Ninety-four rats were randomly assigned into the following five groups: sham, sham + 3-MA (Fluo), SAH + vehicle, SAH + Fluo group, and SAH + Fluo + 3-MA group. PI staining, western blot, and brain water content were measured at 24 h after SAH. Neurological scores were also blindly assessed at the same time.

### SAH model

The SAH model was induced by endovascular perforation as previously described [9], with some modification. Briefly, under pentobarbital (50 mg/kg) anesthesia, the left carotid artery and its branches were exposed and separated. Subsequently, the left external carotid artery (ECA) was cut, and a 4-0 monofilament suture was advanced into the internal carotid artery (ICA) through ECA until resistance was felt. Then, the suture was inserted further to puncture the vessel and induce SAH. The sham rats underwent the same procedure but without vessel puncture.

### Immunofluorescence staining

Propidium iodide (PI; sigma) was dissolved in 0.9% NaCl, and 10 mg/kg PI was administered intraperitoneally at 1 h prior to sacrifice. Brain coronal slices were obtained according to our previous protocol [23]. The slices were incubated at 4 °C over night with mouse monoclonal anti-neuronal nuclei (NeuN) (1:200, MAB377, Millipore), goat polyclonal anti-caspase-1 (1:200, sc-22165, Santa Cruz), and rabbit monoclonal anti-LC3 (1:200, 3868#, Cell signaling Technology) followed by incubation with appropriate secondary antibody (1:200, Jackson ImmunoResearch). Double immunostaining was performed to identify the relation between pyroptosis and autophagy. The primary antibodies that were used were the rabbit monoclonal anti-LC3 (1:200, 3868#, Cell signaling Technology) and goat polyclonal anti-caspase-1 (1:200, sc-22165, Santa Cruz). The secondary antibodies that were used were rhodamine-conjugated donkey anti-rabbit antibody (1:200, Jackson ImmunoResearch) and fluorescein isothiocyanate-labeled donkey anti-goat antibody (1:200, Jackson ImmunoResearch). The sections were blindly observed using a fluorescence microscope.

### Transmission electron microscopy

Rats were sacrificed under deep anesthesia by cardiac perfusion with 0.9% saline and 4% paraformaldehyde in 0.1 M PBS. We obtained the 1-mm^3^ brain slices from the left parietal cortex; samples were then transferred into 2.5% glutaraldehyde and kept overnight at 4 °C. Samples were rinsed several times with buffer and fixed with 1% osmium tetroxide for 1 h. After rinsing again with the distilled water several times, the samples were dehydrated with a series of graded ethanol. Next, infiltration was implemented using a solution of propylene oxide and resin (1:1). Samples were then embedded in resin the next day. 100 nm sections were cut and 4% uranyl acetate (20 min) and 0.5% lead citrate (5 min) were used for staining. A transmission electron microscope was used to examine the cortex ultrastructure.

### Drug administration

N-Ac-Tyr-Val-Ala-Asp-chloromethyl ketone (Ac-YVAD-CMK, Cayman Chemical Company) was dissolved in DMSO and was then diluted in PBS, and injected intraperitoneally (5 mg/kg) 1 h after the induction of SAH as previously described [24]. Fluoxetine was purchased from Selleckchem and dissolved in 0.9% NaCl. Fluoxetine (10 mg/kg) or vehicle was administered intravenously at 6 h after SAH induction as previously described [25]. For the mechanism study, rats were fastened to a stereotactic frame and treated with 5-μl intracerebral ventricular injection of autophagy inhibitor 3-methyladenine (3-MA; 400 nmol) 20 min before SAH onset. 3-MA was purchased from Sigma-Aldrich. Coordinates for intracerebral ventricular injection were 0.8 mm posterior to anterior bregma, 1.0 mm mid to lateral, and 3.8 mm dorsal to ventral. The dose of 3-MA and the coordinates of intracerebral ventricular injection were chosen according to the procedure used in our previous study [26]. After the injection, the needle was left in the site for 5 min before removal and the hole was filled with bone wax.

### SAH grade

The degree of SAH was blindly evaluated as described by Sugawara et al. [27]. In brief, the basal cistern was divided into six parts. Based on the amount of blood in the subarachnoid space, each part was given a score from 0 to 3 and SAH grade was calculated as the sum of each score.

### Neurological scores

The modified Garcia score system was blindly performed to assess the neurological function after SAH [27]. The neurological scores were calculated from a sum of scores from six categories as follows: spontaneous activity, spontaneous movements of all limbs, movements of forelimbs, climbing the wall of the cage, reaction to touch on both sides of the trunk, and response to vibrissae touch. The maximum obtainable score is 18.

### Brain water content

Rats were decapitated under deep pentobarbital anesthesia. Subsequently, the left hemispheres were removed and immediately weighed to obtain the wet weight and then dried at 100 °C for 48 h to obtain the dry weight as previously described [9]. The brain water content was calculated as (wet weight−dry weight)/wet weight × 100%.

### Western blot

Western blot was performed according to our previously protocols [28]. Briefly, the brain tissue was homogenized and centrifuged. The detergent compatible protein assay kit (Bio-Rad, Hercules, CA, USA) was used to determine the protein content. Equal amounts of protein (60 μg) were loaded into the wells of the SDS-PAGE gel. After electrophoresis, the protein was transferred to a nitrocellulose membrane. The membrane was blocked for 2 h using a nonfat dry milk buffer and incubated overnight at 4 °C with the primary antibodies. The following primary antibodies were used: rabbit polyclonal anti-beclin 1 (1:800, ab62557, Abcam), rabbit monoclonal anti-NLRP3(1:1000, ab210491, Abcam), goat polyclonal anti-caspase-1 (1:2000, sc-22165, Santa Cruz), rabbit polyclonal anti-IL-1ß (1:200, ab7973, Abcam), rabbit polyclonal anti-IL-18 (1:1000, cst# 9272, Cell Signaling Technology), and β-actin (1:4000, sc-47778Santa Cruz). The membrane was incubated for 1 h with secondary antibodies (horseradish peroxidase-conjugated) at room temperature. The membrane was exposed to X-ray film, and band densities were analyzed using Image J software. To facilitate comparisons between groups, band density values were normalized to the mean value for the sham group.

### Statistical analysis

The average band density for the control group was set at 1.0, and all band density values were normalized by the average value of the control group. Statistical analyses for two sets of data were performed using Student’s *t* test. A one-way analysis of variance (ANOVA) followed by a Tukey’s multiple comparisons test was used to analyze data for multiple groups. The comparisons of behavior and activity scores were analyzed using the Mann-Whitney *U* test. The data were represented as the mean ± SD. *P* < 0.05 was used for statistical significance.

## Results

### Mortality

The physiological parameters, such as the mean blood pressure and blood gases, were observed, and these parameters were not significantly different between the groups (data not shown). None of the rats died in sham, sham + AC-YVAD-CMK, sham + Fluo, and sham + 3-MA group. In experiment 1, the mortality of the SAH group was 31.6% (12 of 38 rats). In experiment 2, the mortalities were 30.8% (4 of 13 rats) and 25% (3 of 12 rats) in the SAH + vehicle group and the SAH + AC-YVAD-CMK group, respectively. In experiment 3, the SAH + vehicle group exhibited 33.3% (9 of 27 rats) mortality, whereas 21.7% (5 of 23 rats) mortality occurred in SAH + fluoxetine group. In experiment 4, the mortality reached 30.4% (7 of 23 rats), 25% (5 of 20 rats) in the SAH + fluoxetine group, and 28.6% (6 of 21 rats) in the SAH + fluoxetine + 3-MA group. There were no significant differences in mortality between the groups (data not shown).

### Caspase-1 activation occurred in the early stages and accompanied with increased neural necrotic cell death after SAH

**Fig. 1:**
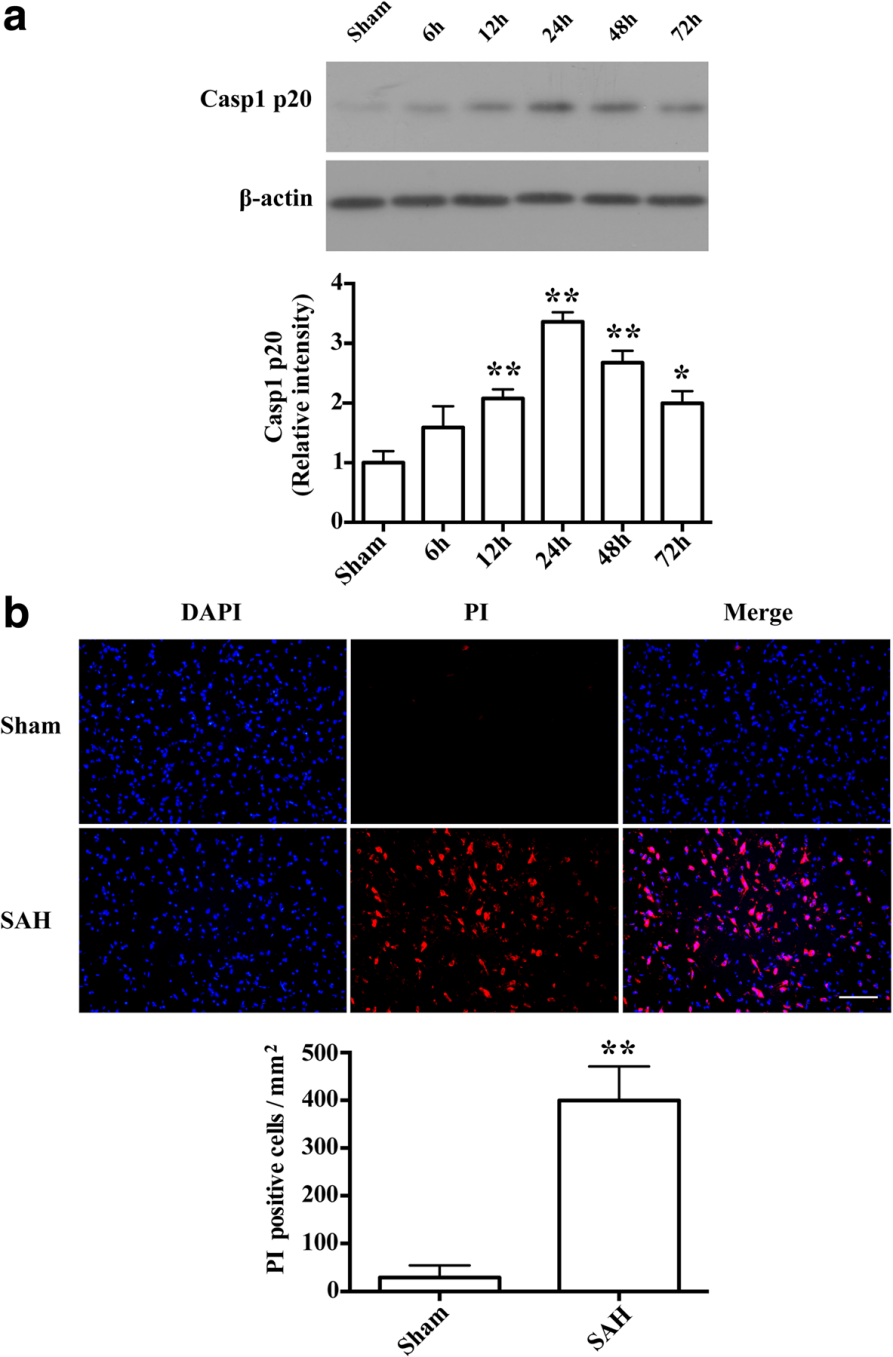
Caspase-1 activation and necrotic cell death were induced in the early stages after SAH. **a** Representative western blot and the relative band densities of caspase-1 p20 in the ipsilateral cortex at different time points (6, 12, 24, 48, and 72 h) after SAH (*n* = 4/group). The bar represents the mean ± SD. *P < 0.05 vs sham, **P < 0.01 vs sham. **b** Representative photomicrographs and the quantification of the PI-positive neural cells in the ipsilateral cortex at 24 h after SAH (*n* = 3/group). Fluorescence colors: DAPI: blue and PI: red. Scale bar = 100 μm, the quantification of the PI-positive neural cells expressed as positive cells per square millimeter. The bar represents the mean ± SD. **P < 0.01 vs sham

**Fig. 2:**
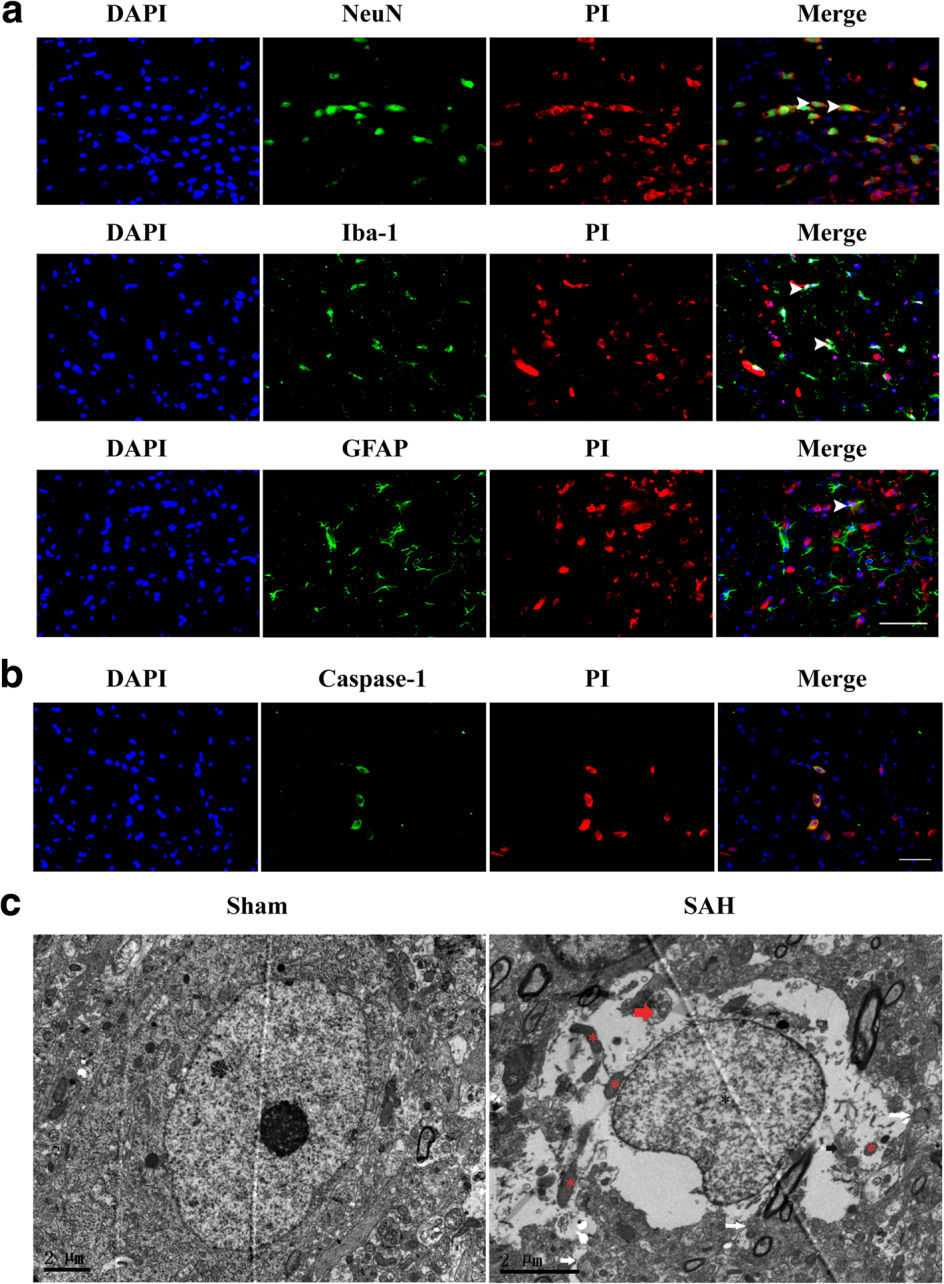
Double staining for PI with NeuN (neuron marker), Iba-1(microglia marker), GFAP(astrocyte marker), and caspase-1, and ultrastructural changes of necrotic cells. **a** Double staining for PI with NeuN (neuron marker), Iba-1(microglia marker), and GFAP(astrocyte marker). Scale bar = 50 μm. **b** Double staining for PI with caspase-1 in the ipsilateral cortex at 24 h after SAH. Scale bar = 50 μm. **c** Ultrastructural changes of necrotic cells at 24 h after SAH. Neurons in the left panel appeared normal with nuclei, mitochondria, and cytomembranes. Neurons in the right panel showed fragmented nuclei (black asterisk), swollen mitochondria (red asterisk), disrupted cytomembranes (white arrow), damaged organelles (white arrow), and double-membrane autophagosomes (red arrow). Scale bar = 2 μm

Western blot results showed that caspase-1 expression levels were significantly increased at 12 h and peaked at 24 h after SAH (*P* < 0.01; Fig. 1a). After peaking, caspase-1 expression levels were declined, but were still high at 72 h post-SAH when compared with the sham group (*p* < 0.05; Fig. 1a). In addition, compared with the sham group, the number of PI-positive cells markedly increased in the SAH group at 24 h after SAH (*P* < 0.01; Fig. 1b). Double immunostaining showed that most of the necrotic cell death was found in neurons, while some were found in microglia and astrocyte (Fig. 2a). Meanwhile, we found that cleaved caspase-1 expression in the necrotic neural cell (Fig. 2b). We further confirmed this caspase-1 antibody did not detect the pro-form of caspase-1 (Additional file 2: Figure S2). Meanwhile, we identified the ultrastructural features of necrotic cells using electron microscopy. As represented in Fig. 2c, neurons in the sham cortex appeared normal with a nucleus, mitochondria, and cytomembranes. Neurons from SAH rats showed a fragmented nucleus (black asterisk), swollen mitochondria (red asterisk), disrupted cytomembranes (white arrow), and damaged organelles (black arrow). Interestingly, we observed double-membrane autophagosome in neurons from SAH rats (red arrow).

### Caspase-1 activation contributes to early brain injury after SAH

**Fig. 3:**
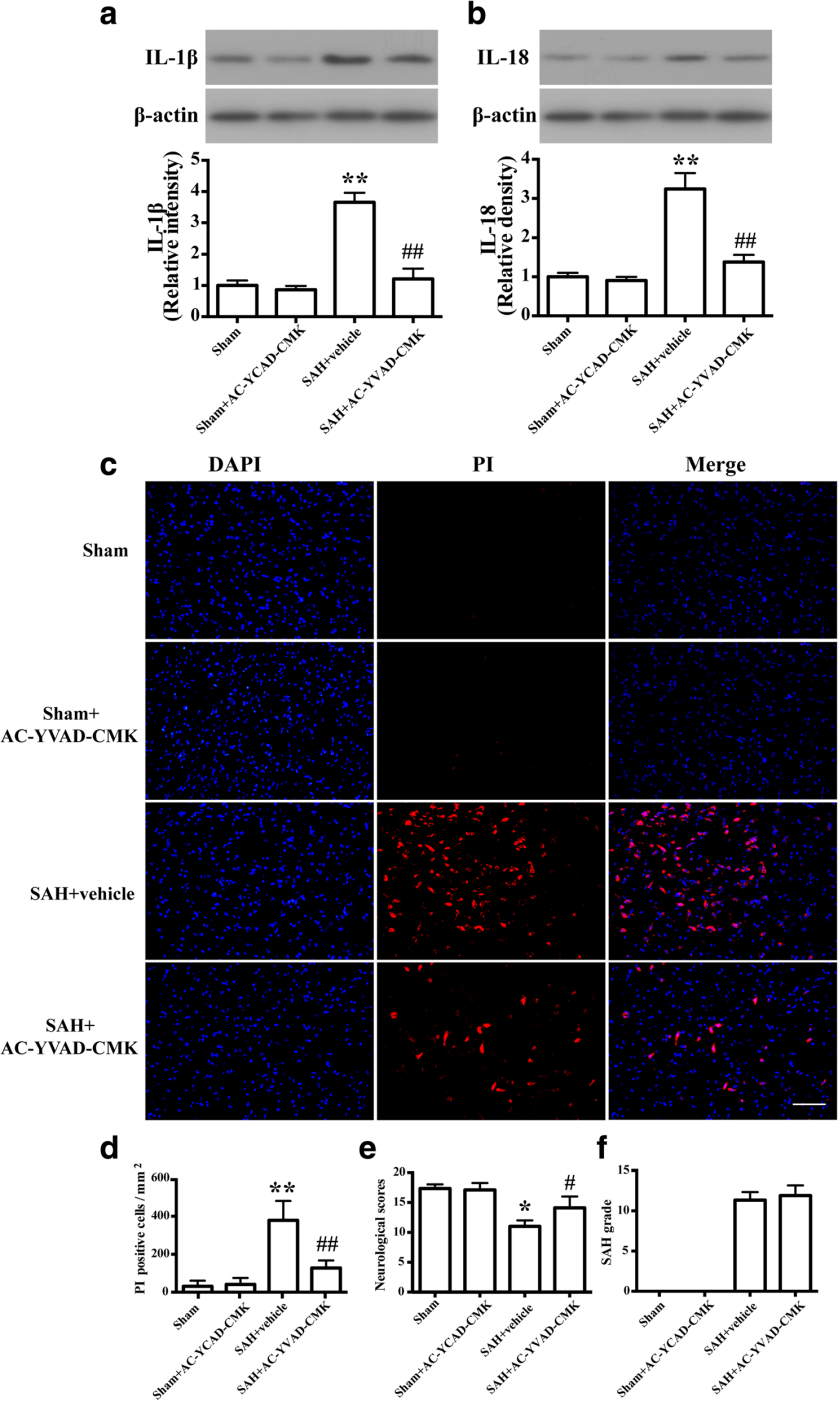
Caspase-1 activation contributes to early brain injury after SAH. **a** Representative western blots and densitometric analyses showing levels of IL-1ß. The bar represents the mean ± SD. **P < 0.01 vs sham. ^##^ *P* < 0.01 vs vehicle control. **b** Representative western blots and densitometric analyses showing levels of IL-18 (*n* = 6/group). The bar represents the mean ± SD. **P < 0.01 vs sham. ^##^ *P* < 0.01 vs vehicle control. **c** Representative photomicrographs of PI staining of neural cells in different groups at 24 h after SAH (*n* = 3/group). Fluorescence colors: DAPI: blue and PI: red. Scale bar = 100 μm. **d** The quantification of the PI-positive neural cells. The quantification of the PI-positive neural cells expressed as positive cells per square millimeter. The bar represents the mean±SD. **P < 0.01 vs sham, ^##^ *P* < 0.01 vs vehicle control. **e** Quantification of neurological scores (*n* = 9). The bars represent the mean with interquartile range. *P < 0.05 vs sham. ^#^ *P* < 0.05 vs vehicle control. **f** Quantification of SAH severity (*n* = 9). The bars represent the mean ± SD. No significant difference was observed between vehicle and SAH+AC-YVAD-CMK group

Western blot results showed that IL-1ß and IL-18 levels were significantly elevated when compared with the sham group at 24 h after SAH (*P* < 0.01, Fig. 3a, b). The protein levels of IL-1ß and IL-18 were significantly decreased by Ac-YVAD-CMK administration (*P* < 0.01; Fig. 3a, b). PI staining showed that PI-positive neural cells were significantly increased in the SAH group when compared with the sham group (*P* < 0.01; Fig. 3c, d). Administration of Ac-YVAD-CMK resulted in a significant reduction of PI-positive neural cells compared with the SAH group (*P* < 0.01; Fig. 3c, d). To further confirm the protective effect of Ac-YVAD-CMK at the macroscopic level, the neurological functions were measured. Consistent with the above results, severe neurological deficits were found in the SAH group compared with the sham group (*P* < 0.05; Fig. 3e), which were improved by AC-YVAD-CMK administration (*P* < 0.05; Fig. 3e). In addition, there was no significant difference between the SAH group and the SAH + AC-YVAD-CMK group (*P* > 0.05; Fig. 3f).

### Fluoxetine attenuates NLRP3 inflammation activation and brain edema and improved neurological function at 24 h after SAH

**Fig. 4:**
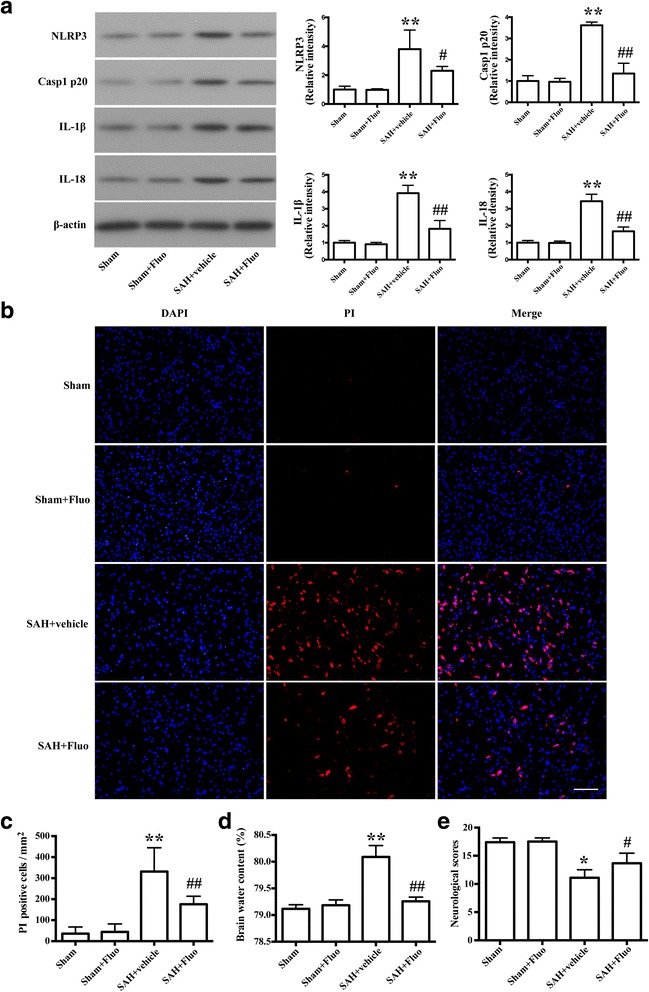
Fluoxetine reduced expression of NLRP3, caspase-1 p20, IL-1ß, and IL-18, decreased the numbers of necrotic cells and brain edema, and improved the neurological scores. **a** Representative Western blots and densitometric analyses of levels of NLRP3, caspase-1 p20, IL-1ß, and IL-18. The bar represents the mean ± SD. **P < 0.01 vs sham. ^#^ *P* < 0.05 vs vehicle control, ^##^ *P* < 0.01 vs vehicle control. **b** Representative photomicrographs of PI-positive neural cells in different groups at 24 h after SAH (*n* = 3/group). Fluorescence colors: DAPI: blue and PI: red. Scale bar = 100 μm. **c** The quantification of the PI-positive neural cells. The quantification of the PI-positive neural cells expressed as positive cells per square millimeter. The bar represents the mean ± SD. **P < 0.01 vs sham, ^##^ *P* < 0.01 vs vehicle control. **d** Quantification of brain water content (*n* = 6). The bars represent the mean ± SD. **P < 0.01 vs sham control, ^##^ *P* < 0.01 vs vehicle control. **e** Quantification of neurological scores (*n*=18). The bars represent the mean with interquartile range. *P < 0.05 vs sham control, ^#^ *P* < 0.05 vs vehicle control

Western blot analysis revealed the expression of NLRP3, cleaved caspase-1, IL-1β and IL-18 were significantly increased compared with the sham rats (*P* < 0.01; Fig. 4a). Fluoxetine administration significantly reduced expression of NLRP3 (*P* < 0.05; Fig. 4a), cleaved caspase-1(*P* < 0.01; Fig. 4a), IL-1β (*P* < 0.01; Fig. 4a), and IL-18 (*P* < 0.01; Fig. 4a). Meanwhile, fluoxetine treatment significantly reduced the number of PI-positive neural cells compared to the SAH + vehicle group (*P* < 0.01; Fig. 4b, c). With regard to brain edema in the ipsilateral hemisphere, fluoxetine significantly reduced the SAH-induced brain edema (*P* < 0.01; Fig. 4d). At 24 h after SAH, fluoxetine administration significantly improved the neurological function compared with the SAH + vehicle group (*P* < 0.05; Fig. 4e).

### Neuroprotective effects of fluoxetine on NLRP3 inflammasome involve autophagy activation after SAH

**Fig. 5:**
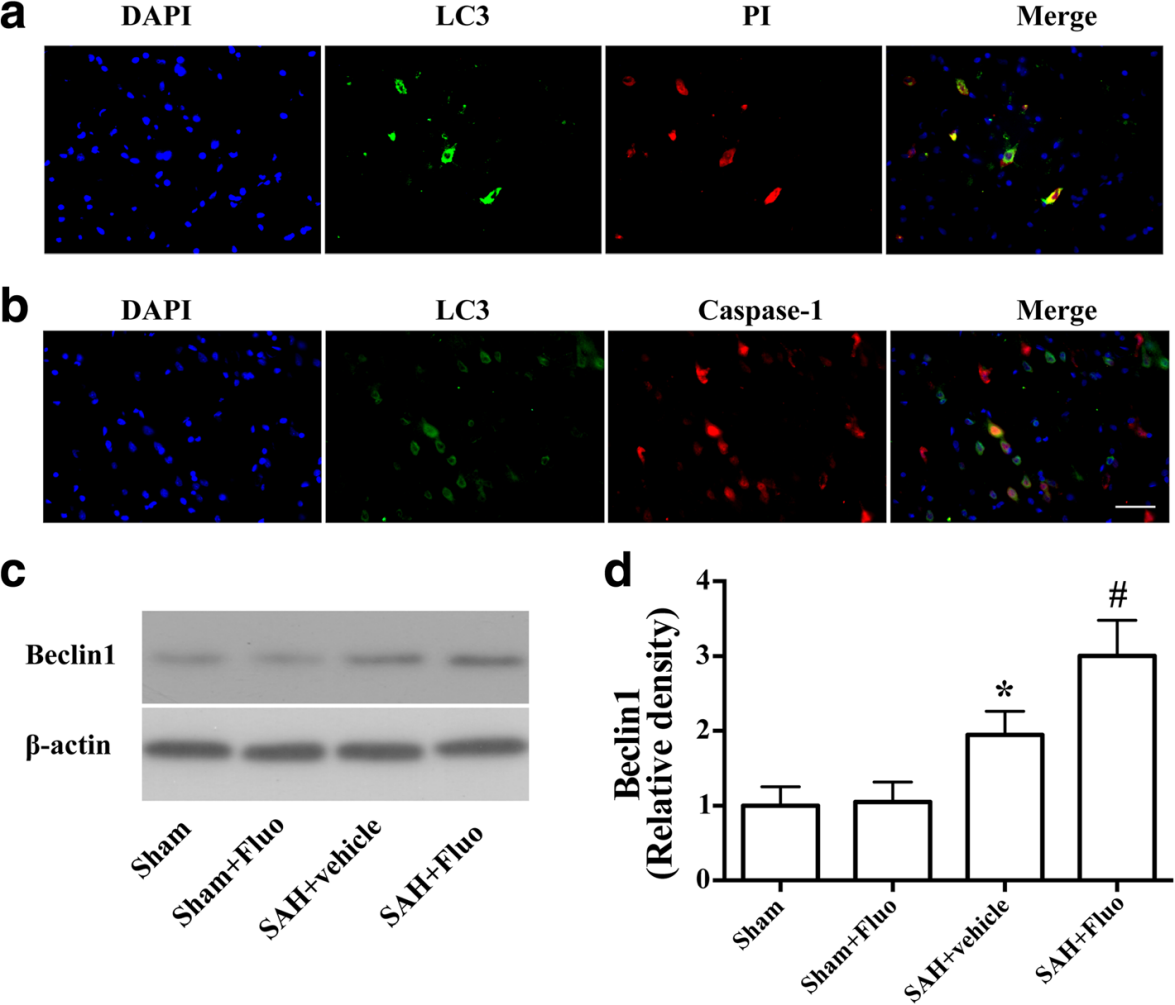
Autophagy activation was involved in the protective effect of fluoxetine on NLRP3 inflammasome activation. **a** Double fluorescence of PI staining (red) and cells with autophagy marker LC3 (green). **b** Representative micrographs showing double immunofluorescence with caspase-1 (red) and LC3 (green). Scale bar = 50 μm. **c** Representative Western blots and **d** densitometric analyses of levels of beclin-1 (*n* = 6). The bars represent the mean ± SD. *P < 0.05 vs sham control, ^#^ *P* < 0.05 vs vehicle control

Double immunostaining showed that LC3 expressions were observed in PI-positive neural cells (Fig. 5a) and co-localized with cleaved caspase-1 in the cortex at 24 h after SAH (Fig. 5b). To further evaluate the autophagy activation, we examined the levels of the autophagy-related protein, becline 1. Western blot results showed that fluoxetine administration upregulated becline 1 levels (*P* < 0.05; Fig. 5c), indicating autophagy activation.

### Autophagy inhibitor 3-MA blocks the effects of fluoxetine on NLRP3 inflammasome activation and brain edema, aggravating neurological deficits after SAH

**Fig. 6:**
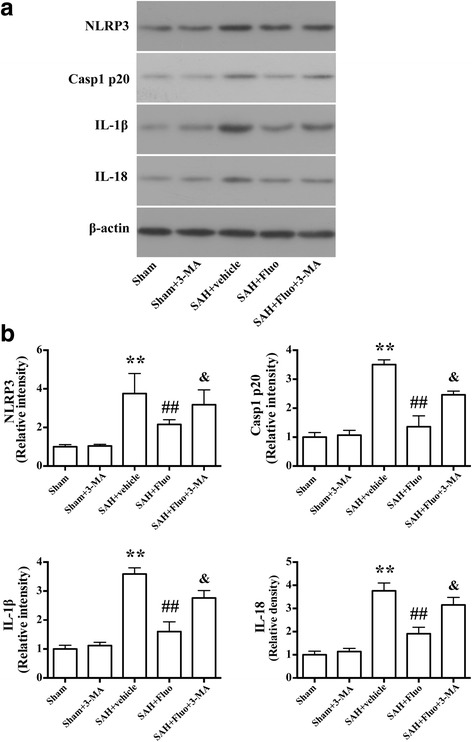
Autophagy inhibitor 3-MA blocks the effects of fluoxetine on expression of NLRP3, caspase-1 p20, IL-1β, and IL-18 after SAH induction. **a** Representative Western blots of NLRP3, caspase-1 p20, IL-1β, and IL-18. **b** Densitometric analyses of levels of NLRP3, caspase-1 p20, IL-1β, and IL-18. *N* = 6. The bar represents the mean ± SD. **P < 0.01 vs the sham group. ^##^ *P* < 0.01 vs SAH + vehicle group. ^&^ *P* < 0.05 vs SAH + fluo group. Representative photomicrographs and the quantification of the PI-positive neural cells in different groups at 24 h after SAH (*n* = 3/group). Fluorescence colors: DAPI: blue and PI: red. Scale bar = 100 μm, the quantification of the PI-positive neural cells expressed as a percent of the total DAPI^+^ cells. The bar represents the mean ± SD. **P < 0.01 vs SAH + vehicle group. ^##^ *P* < 0.01 vs SAH + fluoxetine group

**Fig. 7:**
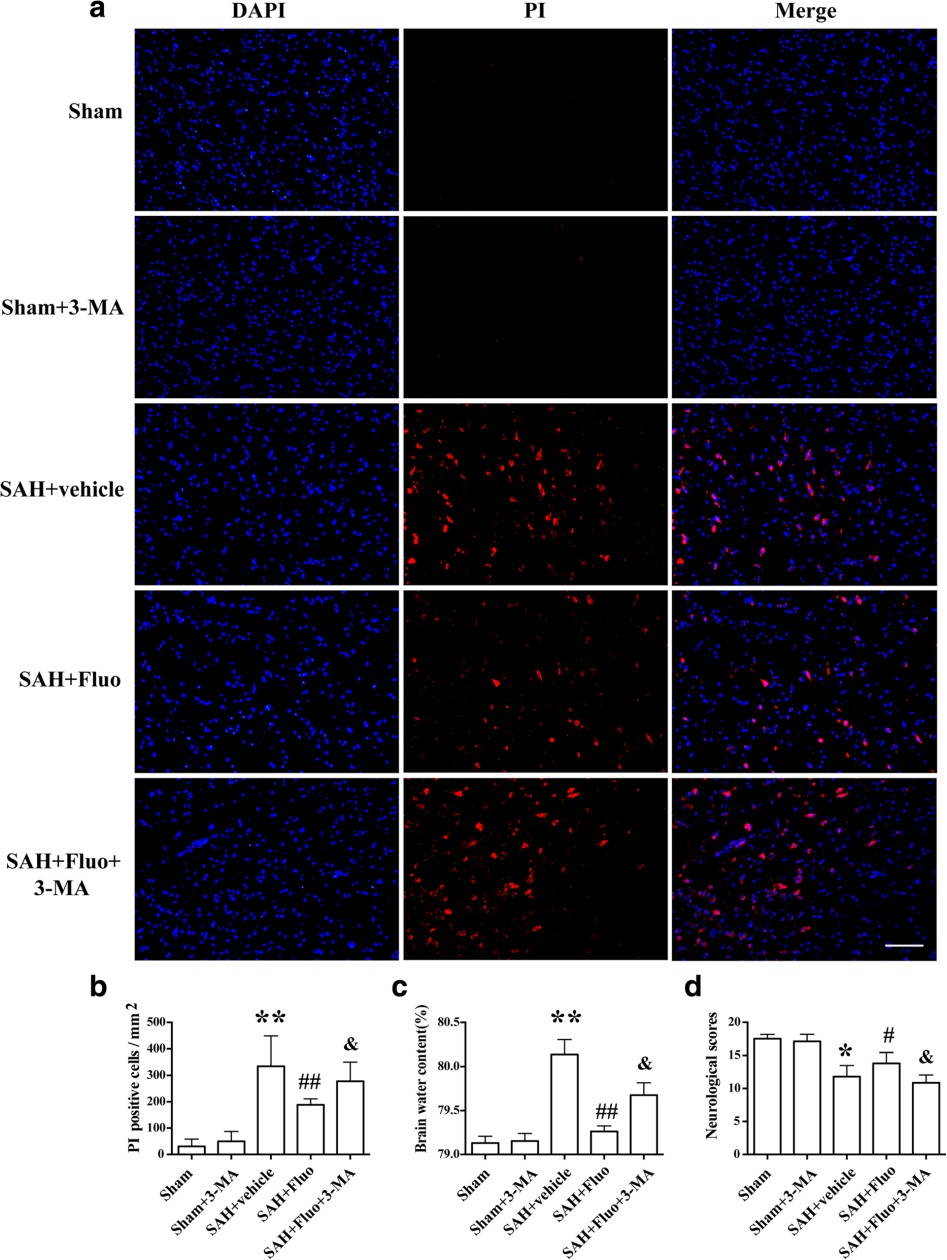
Autophagy inhibitor 3-MA abolishes the neuroprotective effects of fluoxetine on necrotic cell death, brain edema, and neurological function after SAH. **a** Representative photomicrographs of the PI-positive neural cells in different groups at 24 h after SAH (*n* = 3/group). Fluorescence colors: DAPI: blue and PI: red. Scale bar = 100 μm. **b** The quantification of the PI-positive neural cells expressed as positive cells per square millimeter. **P < 0.01 vs the sham group. ^##^ *P* < 0.01 vs SAH + vehicle group. ^&^ *P* < 0.05 vs SAH + fluo group. **c** Quantification of brain water content (*n* = 6). The bars represent the mean ± SD. **P < 0.01 vs the sham group. ^##^ *P* < 0.01 vs SAH + vehicle group. ^&^ *P* < 0.05 vs SAH + fluo group. **d** Quantification of neurological scores (*n* = 15/group). The bars represent the mean with interquartile range. *P < 0.05 vs the sham group. ^#^ *P* < 0.05 vs SAH + vehicle group. ^&^ *P* < 0.05 vs SAH + fluo group

To confirm autophagy activation in the neuroprotective effect of fluoxetine, autophagy inhibitor 3-MA was used. Western blot results showed that co-administration of fluoxetine and 3-MA significantly increased the levels of NLRP3, cleaved caspase-1, mature IL-1ß, and IL-18 compared with the SAH + fluoxetine group (*P* < 0.05; Fig. 6a, b). Notably, immunostaining results showed that co-administration of fluoxetine and 3-MA significantly increased the number of PI-positive neural cells compared to the SAH + fluoxetine rats (*P* < 0.01, Fig. 7a, b). With regard to brain edema in the ipsilateral hemisphere, co-administration of fluoxetine and 3-MA significantly augmented the increased brain edema (*P* < 0.05; Fig. 7c) and worsened the neurological function (*P* < 0.05; Fig. 7d) compared with the SAH + fluoxetine group.

## Discussion

The present study provided the evidence that caspase-1 activation-induced necrotic neural cell death and inflammatory cytokines IL-1β and IL-18 contributed to the early brain injury after SAH. In addition, our report found that fluoxetine attenuated NLRP3 inflammasome and caspase-1 activation and subsequent inflammatory cytokines expression as well as necrotic cell death, reduced the brain edema, and improved neurological deficits. Moreover, autophagy activation was involved in these beneficial effects of fluoxetine, which were abolished by autophagy inhibitor 3-MA. Taken together, our findings suggested that fluoxetine administration, targeting NLRP3 inflammasome and caspase-1 activation, might be an effective therapeutic strategy after SAH.

Caspase-1 is an inflammatory caspase that is well known for the function of cleaving the preforms of inflammatory cytokines IL-1β and IL-18 into their active forms [29]. Both IL-1β and IL-18 are the important inflammatory mediator in early brain injury after SAH. These inflammatory cytokines induced matrix metalloproteinase (MMP)-9 expression and disruption of the blood brain barrier (BBB), aggravating brain edema and neurological deficits after SAH [24]. Clinical studies have demonstrated that caspase-1 and subsequent inflammatory cytokines elevated in cerebrospinal fluid (CSF) of patients with SAH [30, 31]. In the current study, we found that cleaved caspase-1 was increased and peaked at 24 h after SAH. Besides, increasing studies indicated that caspase-1 also induced the necrotic cell death by cleaving adermin-D (GSDMD), which lyses the liposomes and forms the pores on the membranes [32–34]. Our study indicated that caspase-1 activation was accompanied with increased necrotic neural cells. In addition, most of these necrotic neural cells were neurons, which is consistent with previous studies [35]. And some of them were microglia and astrocytes. Additionally, the ultrastructural features of necrotic cell death were identified by using a transmission electron microscope. DNA fragmentation, swollen mitochondria, damaged cytoplasmic organelles, cytoplasmic hyper-vacuolization, and cytomembrane rupture were observed. Intriguingly, autophagic vacuoles were also observed, indicating that autophagy may be involved in the pathology of caspase-1-induced necrotic cell death. These features are consistent with previous studies [36, 37]. Caspase-1 inhibitor AC-YVAD-CMK downregulated the expression of mature IL-1β and IL-18, reduced the number of necrotic cell death, and improved neurological function in SAH rats. Taken together, our study indicated that caspase-1 activation contributes to the early brain injury after SAH.

Caspase-1 activation requires a multiprotein platform, namely inflammasome. To date, NLRP1b, NLRP3, and NLRC4, as well as the cytosolic DNA sensor absent from in melanoma2(AIM2), are well-defined inflammasomes [38]. Our previous studies demonstrated NLRP3 inflammasome contribute to the neuroinflammation in early brain injury after SAH [9, 14]. A recent clinical study demonstrated NLRP3 inflammasome and caspase-1 were elevated in CSF of SAH patients and associated with functional outcome of these patients [30]. Therapies targeting NLRP3 inflammasome activation attenuated early brain injury and improved neurological function after SAH in rats [9, 10, 39]. Traditionally, fluoxetine is a common treatment for major depression due to its safer profile, greater tolerability, and fewer side effects. Recently, the protective pharmacological effects of fluoxetine have been demonstrated in clinical practice in the treatment of neurological diseases, such as ischemic [18], Alzheimer’s disease [40], and traumatic brain injury [41]. Emerging evidence suggests that fluoxetine has anti-inflammatory properties [42, 43]. But the precise mechanism is uncertain. In our present study, fluoxetine was found to reduce the expression of NLRP3, cleaved caspase-1, IL-1β, and IL-18, alleviate the neural necrotic cell death and brain edema, and improve the neurological function after SAH. These data indicated that fluoxetine alleviates early brain injury after SAH through the inhibition of NLRP3 inflammasome activation.

Autophagy is an intracellular process for cellular homeostasis and recycling of damaged organelles and proteins, as well as the destruction of intracellular pathogens [44]. Activated autophagy pathway has been demonstrated in experimental SAH [26, 45, 46]. More and more studies have demonstrated the regulatory roles of autophagy in inflammasome activation. The loss of proteins that are essential for autophagy activation, such as Atg16L1 and Atg7, resulting in caspase-1 activation as well as increased production of IL-1β and IL-18 in macrophages [47, 48]. In addition, autophagy can also negatively regulate inflammasome activation through removing damaged mitochondria to preventing the release of mtROS and mtDNA into the cytoplasm, and ultimately limiting inflammasome assembly [49, 50]. Additionally, assembled inflammasomes can be degraded by autophagosomes through the autophagic protein p62 [51, 52]. Fluoxetine upregulated the expression of beclin-1, which is compatible with previous foundings that fluoxetine activates autophagic pathways via FKBP51 [21]. Moreover, autophagy inhibitor 3-MA reversed the neuroprotective effects of fluoxetine in our present study. These data suggest that fluoxetine provides potential therapeutic interventions for EBI after SAH through the inhibition of NLRP3 inflammasome activation by enhancing autophagy.

It is important to note that there are several limitations in our present study. First, the antiapoptotic and antioxidant property of fluoxetine has been described; therefore, we cannot exclude the possibility that these properties also play a role in the neuroprotective effect of fluoxetine. Second, our study showed that caspase-1 activation induced neural necrotic cell death, but whether these necrotic cell death are pyroptosis needs further study. In addition, we studied the short-term effects of fluoxetine after SAH, but the side-effects of fluoxetine, such as myeloid response, warrant further study.

## Conclusion

The present study demonstrated that NLRP3 inflammasome and caspase-1 activation contributed to early brain injury after experimental SAH. Fluoxetine inhibits NLRP3 inflammasome activation and subsequent caspase-1 activation and reduces early brain injury after SAH, at least partly, by activating autophagy, providing potential therapeutic interventions for SAH.

## Additional files

Additional file 1: Figure S1. The study design. (TIFF 1720 kb)

Additional file 2: Figure S2. Western blot figure that shows the caspase-1 antibody used in the IHC did not detect the pro-form of caspase-1. (TIFF 2497 kb)

## Abbreviations

3-MA: 3-Methyladenine
Ac-YVAD-CMK: N-Ac-Tyr-Val-Ala-Asp-chloromethyl ketone
BBB: Blood brain barrier
CBF: Cerebral blood flow
CCP: Cerebral perfusion pressure
CSF: Cerebrospinal fluid
ECA: External carotid artery
FKBP51: FK506 binding protein 51;
GSDMD: Gasdermin-D
ICA: Internal carotid artery
ICP: Intracranial pressure
MMP: Matrix metalloproteinase
SAH: Subarachnoid hemorrhage
SD: Sprague-Dawley
SSRI: Selective serotonin reuptake inhibitor
