# Supplementary figures and images for: Fluoxetine-enhanced autophagy ameliorates early brain injury via inhibition of NLRP3 inflammasome activation following subrachnoid hemorrhage in rats
Source: J Neuroinflammation. 2017 Sep 13;14:186. doi: 10.1186/s12974-017-0959-6 (PMC5598033; doi:10.1186/s12974-017-0959-6)

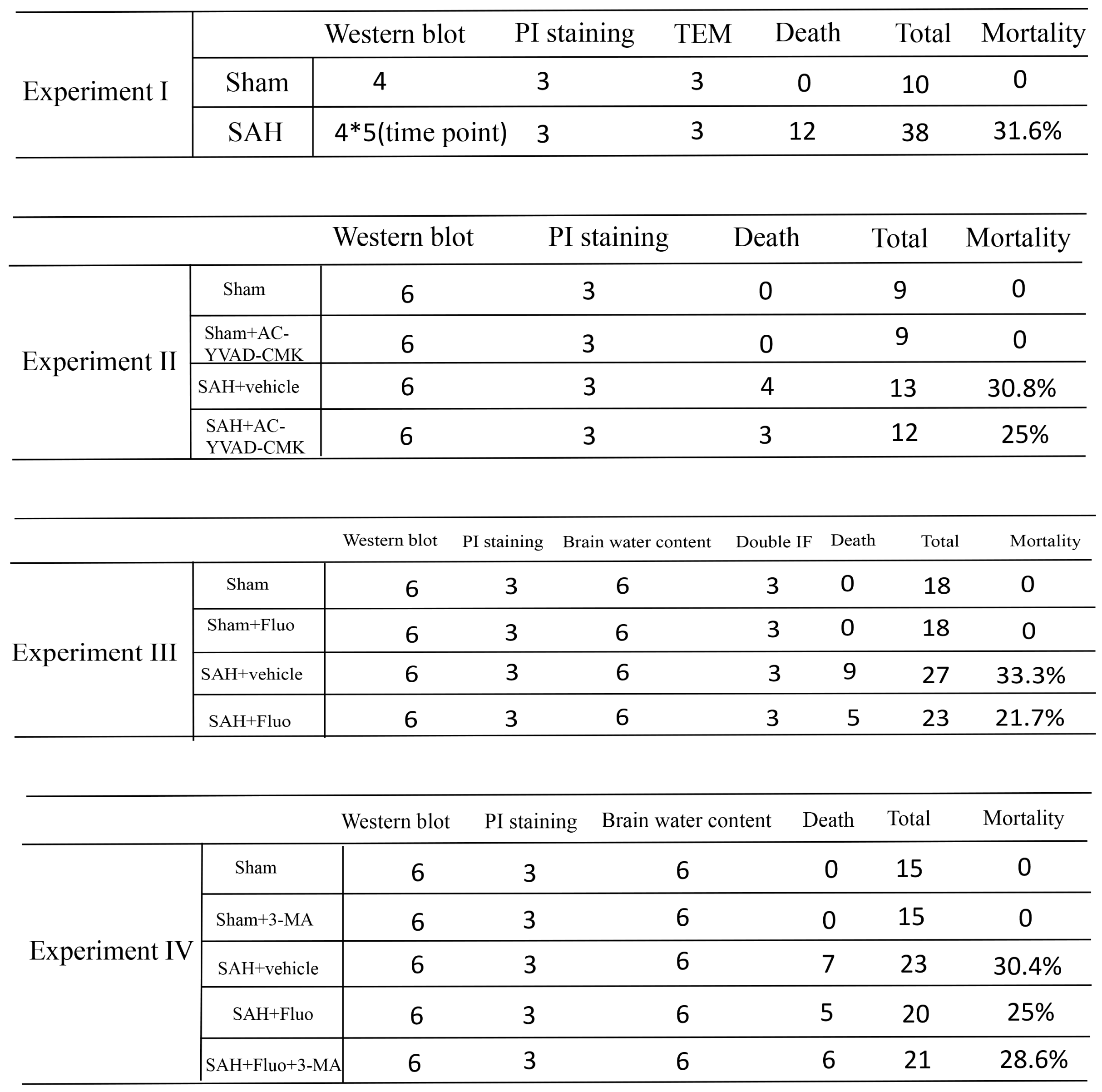

Supplement: Supplementary file 1 — The study design. (TIFF 1720 kb) [file 12974_2017_959_MOESM1_ESM.tif]

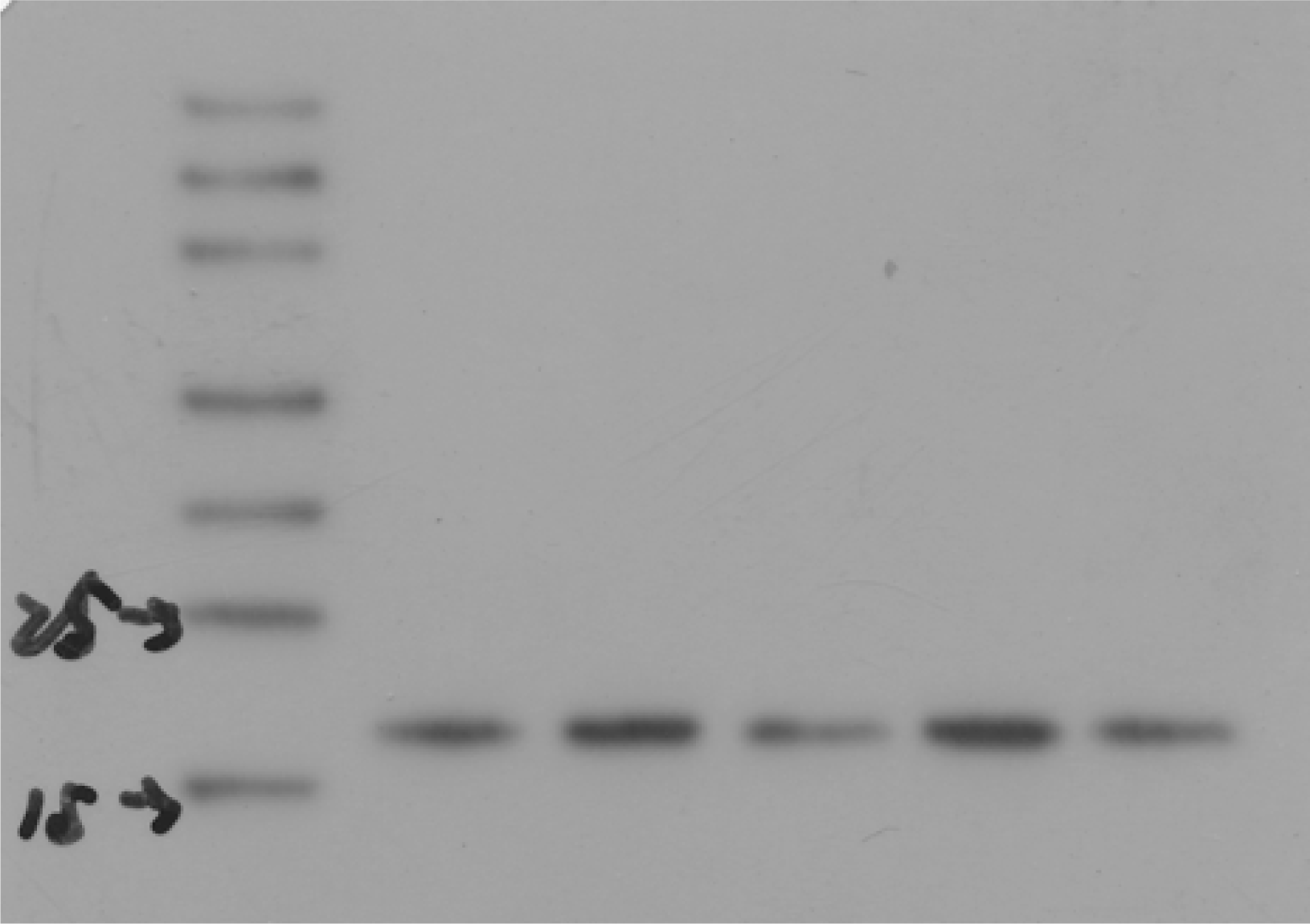

Supplement: Supplementary file 2 — Western blot figure that shows the caspase-1 antibody used in the IHC did not detect the pro-form of caspase-1. (TIFF 2497 kb) [file 12974_2017_959_MOESM2_ESM.tif]
